# Socio-demographic and water handling practices affecting quality of household drinking water in Kisii Town, Kisii County, Kenya

**DOI:** 10.1016/j.heliyon.2022.e09419

**Published:** 2022-05-14

**Authors:** J.K. Ondieki, D.N. Akunga, P.N. Warutere, Omanga Kenyanya

**Affiliations:** a School of Public Health, Kenyatta University, Kenya; b School of Pure and Applied Sciences, Kisii University, Kenya

**Keywords:** Water quality, Determinants, Socio-demographic, Hygiene and sanitation

## Abstract

Household drinking water quality is dependent on a number of determinants which could be arising at the source, during transportation or due to storage and handling practices. The challenge of unsafe water is even big in urban settings that are often characterized by exponential population growth, increased urbanization, industrialization and poor sanitary facilities. Contaminated water is a leading cause of water borne diseases which are a major public health and policy makers concern. In fact, Water borne diseases are third cause of mortality in Kenya whereas they are ranked second in Kisii. The study was designed to investigate the factors affecting household drinking water quality in Kisii Town that has four main zones which include: Mwembe, Jogoo, Nyanchwa and CBD. Demographics, level of awareness in terms of water quality as well as hygiene and sanitation practices of the study population were studied using questionnaires. The questionnaires were filled by interviewing household heads from 422 sampled households. Qualitative data was also obtained by use of Focused group discussions (FGDs). Perspectives of key people such as public health officers were acquired through Key informant interviews (KIIs). The study found a significant relationship between household size and water quality in terms of presence of total coliforms. The following hygiene and sanitation factors were found to be having significant relationship with presence of *E. coli* in household drinking water; source of water (p = 0.002), transportation container (p = 0.029), covering during transportation (p = 0.012), storage container (p < 0.001), method of drawing from storage container (p < 0.001), feces disposal (p = 0.001) and garbage disposal method (p = 0.04). The conclusion of this study is that good hygiene and sanitation practices are important in ensuring total safety of drinking water at the point of use. There is therefore need for more capacity building in this region to ensure that people do not consume contaminated water which is a major contributing factor to water-borne diseases.

## 1 Introduction

More than 700 million people globally lack access to safe drinking water and close to half of these are from Sub-Saharan Africa (WHO/UNICEF, 2014). According to the Kenyan constitution (Article 43 (1) (d)), every person has a right to clean and safe water in adequate quantities (GOK, 2010). In order to achieve vision 2030 Kenya hopes to sustain the provision of basic services such as safe water and basic sanitation (Onjala et al., 2014).

Approximately, 75% of Kisii town residents are not connected to piped water. Most of the water used within the town for domestic purposes is drawn raw from protected and unprotected sources (Onjala et al., 2014). Majority of the households do not understand the risks associated with the unsafe water from most of these sources which have been found to contain high levels of thermo-tolerant coliforms (Aaron Gichaba Misati et al., 2017; Onjala et al., 2014). World Health Organization (WHO, 2017) recommends household water treatment (HWT) of both improved and unimproved sources, if the water is collected from unreliable piped supplies, non-piped supplies outside the home and unimproved water sources. However, inadequate knowledge, poor attitudes and unhygienic practices in households makes it unlikely that the household water treatment technologies will be effective in reducing microbial concentrations to the standards stipulated by accepted drinking water quality guidelines (Onabolu, 2013).

Even though water source determines the quality of drinking water (Usman et al., 2016), poor water handling practices and low hygiene level have also been linked to post-contamination (Gizachew et al., 2020; Kioko and Obiri, 2012). Utensils used to draw water from the storage container and hands of the water handler can be a source of contamination (WHO, 2017). A study conducted in Kericho, Kenya showed that household practices such as drawing of water, hand-washing, storage type, human waste disposal, water treatment and general cleanliness were correlated with thermocolerant coliforms contamination (Too et al., 2016). Storage containers that require inserting of other smaller containers in order to obtain the water, have been found to cause a lot of contamination (Kirianki et al., 2017). The hygiene of the water handler and the cleanliness of the drawing container influences water quality as these may contain contaminants that could be introduced into the drinking water during fetching at both the source and at household level (Brown et al., 2013). Recontamination of drinking water poses great health risk as it is associated with higher cases of diarrhea especially among infants and immunosuppressed individuals (Trevett et al., 2005). Water borne diseases are third cause of mortality in Kenya whereas in Kisii, it is the second cause of mortality (KCHSIP, n.d.).

Contaminated drinking water at the point of use could be attributed to a number of determinants which could be at the source, during transportation, during storage or at the household. The study was therefore necessary to establish various socio-demographic and water handling factors that are associated with household drinking water quality at the point of use in Kisii Town.

## 2 Literature review

Microbial diseases caused by coliforms are known to be transmitted through food and water (Nkere et al., 2011). Bacteriological parameters specifically total coliforms and *E coli* are commonly used in the general determination of quality of drinking water globally (WHO, 2017). Consumption of water containing excreta from humans and animals poses great risk to public health. This is because of presence of microbes known as fecal coliforms which are subsets total coliforms found in intestines of warm-blooded animals (Tsega et al., 2013).

Quality of drinking water at the point-of-use is dependent on many factors within and around the household. Socio-demographic factors such as age and gender of the household head have been found to be influencing the quality of household drinking quality. Size of the household and level of education can too affect quality of stored water (Usman et al., 2016). In terms of age, it has been revealed that older women have better understanding of water contamination compared younger women because they may have had more contact with health officers (Figueroa and Kincaid, 2010).

Studies have shown that most waters from open sources are not safe for drinking and does not comply with WHO standards hence requires some form of treatment. The sources include open wells, open reservoirs and unprotected springs which have been found to contain great numbers of colony-forming units of *E. coli* and total coliforms (Gwimbi, 2011). Improper disposal of human excreta such as construction of pit latrines too close to water sources and poor protection of water at the source are considered to be major causes of fecal contamination. It has been found that households with exposed excreta, in most cases contain high number microbial contaminants (Marshall, 2011; Tsega et al., 2013). Human feces may contain variety of pathogens which cause diseases such as typhoid, dysentery, cholera and gastro-enteritis (Akpor and Muchie, 2011).

Many studies have been conducted in Kisii County on hygiene and sanitation practices that may affect the quality of drinking water in households such as that of A. G. Misati (2016). Water sampling and analysis has also been conducted on water from various sources by researchers as Kioko and Obiri (2012), Onjala et al. (2014) and Ogendi G.M, A.M., Getabu, J.M. Onchieku, J.M. (2015). Unfortunate, there is no available published literature on direct linkage between water quality at the point of use in households and various socio-demographic factors as well as water-handling practices. A study of this kind was therefore necessary to investigate this linkage.

## 3 Methods

### 3.1 Study area description

Kisii Town located at 0° 41′ 0″ South, 34° 46′ 0″ East is the biggest among all towns of Kisii County and serves as the County headquarters. It is a fast growing town with many upcoming residential and commercial buildings. The residential areas are unequally distributed in the CBD, Jogoo, Nyanchwa and Mwembe strata. It however faces the challenge of waste management as there is no proper designated dumping site and the current site is adjacent a major river.

### 3.2 Research design

Researcher administered questionnaires were used to study the hygiene and sanitation practices in households as well as other factors that could influence household drinking water quality. The data was obtained from 422 household heads, whereby this sample size was arrived at using Fisher's formula for populations over 10000 (Fischer, 1998). As per Kisii County Integrated Development plan 2018–2022, the population of Kisii Town by 2018 was 74098 (CIDP, 2018). To ensure equal distribution of samples within the four strata, proportionate stratified random sampling was employed. Random sampling was used within the strata because the exact number of households was not known. The researcher administered questionnaires were preferred due to varying levels of literacy among the respondents in the study area. Qualitative data was also collected by use of focused group discussions (FGDs) and key informant interviews (KIIs) were used to gather expert opinions. A total of three FGDs were conducted each comprising of eight household heads and the aim of these was to gather further knowledge on the attitudes of the respondents towards their drinking water. The KIIs were administered to two public health officers working in Kisii Town council and two Gusii Water and Sanitation Company (GWASCO) employees. The company is the one that supplies piped water within Kisii Town.

### 3.3 Data analysis and quality control

The data obtained was entered into SPSS software version 20. Both descriptive and inferential analyses were conducted and the data obtained presented in tables and figures. Chi-square tests were used to test association between various parameters. The questionnaires were first tested in Menyinkwa area (a different nearby location) in April 2019 to gauge their suitability after which required changes were made before the real study was rolled out between May–October 2019. Table 1 below shows results of bacteriological quality of drinking water in the studied households as reported earlier by Ondieki et al. (2021). These results were generated from tested water samples that were simultaneously collected with the questionnaires. The main critical parameter of determining drinking water quality in community supplies is *E. coli* which is an indication of feacal contamination and therefore considered as the main dependent variable in this study (WHO, 2017). The study established relationships between drinking water quality and various factors in relation to water handling.

**Table 1:** Summary of prevalence of total coliforms and *E. coli* in different zones of Kisii Town between May–October 2019.

Zone	Sample Size	Total Coliforms	*E. coli*	Non *E. coli*	WHO/KEBS Standards	
					Total Coli forms	*E. coli*
Jogoo	261	101 (38.4%)	47	54	0 CFU	0 CFU
CBD	25	7 (28%)	3	4		
Nyanchwa	32	18 (56.3%)	8	10		
Mwembe	104	41 (39.4%)	16	25		
Total	422	167 (39.6%)	74	93		

(source: Ondieki et al., 2021).

## 4 Results

### 4.1 Socio-demographic and socio-economic characteristics of the respondents

In the study, a total of 422 respondents were interviewed providing an overall response rate of 100%. From the findings, 276 (65.4%) of household heads were male while 146 (34.6%) were females. Out of the 422 respondents, 138 (32.7%) were within 30–39 years, 134 (31.8%) were within 18–29 years, 78 (18.5%) were within 40–49 years, 52 (12.3%) were within 50–59 years and 20 (4.7%) were 60 or more. Most household heads, that is, 269 (63.7%) were married, 115 (27.3%) were single, 23 (5.5%) were widowed, 12 (2.8%) were separated and 3 (0.7%) were divorced. In the study, 203 (48.1%) of the respondents had secondary education, 121 (28.7%) tertiary education, 87 (20.6%) primary education and 11 (2.6) had none. Most households, that is, 241 (57.1%) comprised 5 or less members while 181 (42.9%) had more than 5 members. Table 2 gives summary on socio-demographic characteristics of the studied population.

**Table 2:** Summary of findings of Kisii Town household socio-demographic characteristics between May–October 2019.

Variable	Category	Frequency	Percentage (%)
Gender of household head	Male	276	65.4
	Female	146	34.6
	**Total**	**422**	**100**
Age of the respondent (years)	18–29	134	31.8
	30–39	138	32.7
	40–49	78	18.5
	50–59	52	12.3
	60 and above	20	4.7
	**Total**	**422**	**100**
Marital status	Single	115	27.3
	Married	269	63.7
	Divorced	3	0.7
	Widowed	23	5.5
	Separated	12	2.8
	**Total**	**422**	**100**
Level of education	None	11	2.6
	Primary	87	20.6
	Secondary	203	48.1
	Tertiary	121	28.7
	**Total**	**422**	**100**
Household size	1–5	241	57.1
	More than 5	181	42.9
	**Total**	**422**	**100**

### 4.2 Relationship between socio-demographic characteristics and fecal contamination of drinking water

The comparisons in this and subsequent sections are based on findings published earlier by Ondieki et al. (2021). According to the findings, out of the tested 422 water samples, 167 (39.6%) were contaminated with total coli forms while 74 (17.5%) had *E. coli.*

Pearson correlation was conducted to establish if there existed a relationship between various socio-demographic characteristics and fecal contamination of drinking water. The factors studied include household head, key decision maker concerning drinking water, age of the decision maker, marital status, education level and household size. It was noted that there was no significant relationship observed in all the studied factors as shown in Table 3. Given that the study was carried out in an urban setting, it was noted that female household heads' education levels and economic status do not differ significantly with those of their male counterparts regardless of age and marital status. This could be the reason why there was also no observed association in terms of socio-demographic factors and water quality.

**Table 3:** Relationship between socio-demographic characteristics and fecal contamination of household drinking water in Kisii Town between May–October 2019.

Variable	n = 422	Presence/absence of *E. coli*
Gender of household head	Pearson correation	-0.036
	Sig. (2-tailed)	0.459
Age of household head	Pearson correation	0.036
	Sig. (2-tailed)	0.461
Marital status of household head	Pearson correation	-0.009
	Sig. (2-tailed)	0.860
Level of education of household head	Pearson correation	-0.024
	Sig. (2-tailed)	0.627
Household size	Pearson correation	-0.072
	Sig. (2-tailed)	0.140

### 4.3 Relationship between household size and presence of total coliforms in household drinking water

The study found a significant relationship with presence of total coliforms in household drinking water and household size (p = 0.048). From the odds ratio, households with more than 5 members are 1.5 more likely to have total coliforms in their drinking water when compared with those with 5 or less members. Table 4 illustrates these relationships. The two classifications were arrived at based on a recent study by Armah and others (Armah et al., 2018) whereby small size families are those with 1–5 members, medium size families comprise 6–10 members while large size families comprise of more than 10 members. Given that there were few households with more than 10 members in the current study, this category was incorporated into that of 6–10 members.

**Table 4:** Relationship between household size and presence of total coliforms in household drinking water.

Variable		Presence of total coliforms	Absence of total coliforms	Statistics	P value
Husehold size	1-5 members	85 (35.3%)	156 (64.7%)	χ^2^ = 3.894 df = 1 OR = 1.5	Ρ = 0.048
	More than 5 members	81 (44.8%)	100 (55.2%)		

### 4.4 Household water treatment system (HWTS)

**Figure 1:**
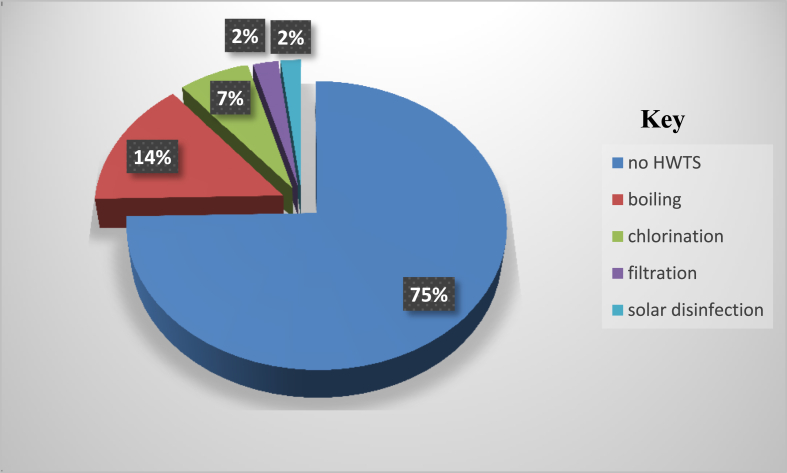
Household water treatment systems (HWTS) in Kisii Town between May–October 2019.

Most of the respondents from the sampled households, 315 (74.6%) were not using any method of water treatment. Sixty-one (14.5%) practiced boiling, 28 (6.6%) used chlorination method, 10 (2.4%) used filtration and 8 (1.9%) used solar disinfection as shown in Figure 1.

### 4.5 Factors influencing household water treatment

Factors that were found to be having a statistically significant relationship with HWTS are source of drinking water (p < 0.001) and water quality perception (p < 0.001). unimproved water source users had a relatively higher proportion (38.1%) when compared to 21.5% of households that obtained water from improved sources who treated their drinking water. majority of households (72.9%) that perceived their water to be unsafe practiced HWTS when compared to their counterparts who perceived their water to be safe whereby only 11.2% from this category practiced the same as shown in Table 5.

**Table 5:** Factors influencing household water treatment in Kisii Town between May–October 2019.

Variable		HWTS	No HWTS	Statistics	P Value
Water source	Improved	70 (21.5%)	255 (78.5%)	χ^2^ = 10.88 df = 1	p = 0.001
	Non-improved	37 (38.1%)	60 (61.9%)		
Water quality perception	Safe	21 (11.2%)	167 (88.8%)	χ^2^ = 90.456 df = 2	p < 0.001
	Not safe	43 (72.9%)	16 (27.1%)		
	Not sure	43 (24.6%)	132 (75.4%)		

### 4.6 Relationships between hygiene and sanitation practices and fecal contamination of drinking water

The study found a strong relationship between fecal contamination and the following factors: water source (p = 0.002), transportation container (p = 0.029), covering during transportation (p = 0.012), storage container (p < 0.001), method of drawing from storage container (p < 0.001), feces disposal (p = 0.001) and garbage disposal method (p = 0.04). Factors that were found to be contributing to fecal contamination of drinking water include: obtaining water from non-improved sources, using wide mouthed container during transportation and storage, not covering the drinking water, inserting another container during drawing, exposed fecal matter (pit latrines) and exposed excreta (throwing in the open or garden). This summary is illustrated in Table 6.

**Table 6:** Relationships between hygiene and sanitation practices and fecal contamination of household drinking water in Kisii Town between May–October 2019.

Variable		Presence of E-coli n (%)	Absence of E-coli n (%)	Statistics	p-Value
Drinking water source	Improved	49 (15.1)	276 (84.9)	χ^2^ = 4.88 df = 1	p = 0.03
	Non-improved	24 (24.7)	73 (75.3)		
Drinking water transportation	Bucket	25 (23.1)	83 (76.9)	χ^2^ = 9.05∗ df = 3	p = 0.029
	Jerry can	47 (17.9)	216 (82.1)		
	Piped	1 (2.7)	36 (97.3)		
	Bottle	1 (7.1)	13 (92.9)		
Covering during transportation	Yes	29 (14.4)	176 (85.6)	χ^2^ = 10.97 df = 3	p = 0.012
	No	27 (20.5)	105 (79.5)		
	Sometimes	17 (28.8)	42 (71.2)		
	No transportation	1 (3.8)	25 (96.2)		
Drinking water storage	Tap	1 (2.7)	36 (97.3)	χ^2^ = 44.26 df = 5	p < 0.001
	Bucket	7 (23.3)	23 (76.7)		
	Clay pot	10 (47.6)	11 (52.4)		
	Jerry cans and bottles	35 (12.4)	247 (87.6)		
	Drums	15 (40.5)	22 (59.5)		
	Tank	6 (42.9)	8 (57.1)		
Drinking water drawing from storage container	By tap	3 (6)	47 (94)	χ^2^ = 45.34 df = 2	p < 0.001
	By pouring	35 (12.2)	251 (87.8)		
	By inserting another container	36 (41.9)	50 (58.1)		
Excreta management	Pit latrine	58 (22.1)	205 (77.9)	χ^2^ = 9.85 df = 1	p = 0.002
	Flash toilet	16 (10.1)	143 (89.9)		
Garbage disposal	Throw in the open	15 (19)	64 (81)	χ^2^ = 8.32 df = 3	p = 0.04
	Household/homestead bin	10 (8.9)	102 (91.1)		
	Public garbage bin	34 (20.4)	133 (79.6)		
	In the garden	15 (23.4)	49 (76.6)		

∗ Fisher's exact test.

Improved water sources included piped water, protected spring, borehole, tube-well, protected dug well and rainwater. On the other hand, unimproved water sources included unprotected spring, unprotected well and vended water usually transported by hand carts (Usman et al., 2016). Pit latrines especially when constructed near water sources introduce fecal matter into the water. This is a common phenomenon in Kisii Town which has experienced rapid population growth in recent years. Exposed garbage harbors houseflies which are the key transmission agents of fecal matter. Water stored in wide mouthed containers such as buckets, clay pots and drums was more contaminated than water stored in jerry cans. This could be because the first category of containers involves dipping other smaller containers in order to obtain the drinking water. These smaller containers could contain contaminants introduced to them from the water handler or from the environment (Kirianki et al., 2017).

### 4.7 Qualitative data results

Results from qualitative data were divided into two main parts. The first part is about the perceived quality of household drinking water while the second part is about factors that could be contributing to poor water quality in the study area.

#### 4.7.1 Perceived water quality

The aspect of water quality perception brought out mixed reactions when it came to FGDs whereby many participants stated that they were not sure about the quality of their drinking water. The main factor that seemed to influence their perception was diarrhea incidences in their households.

***“I am usually not sure of the quality of the water I get from water vendors because I don’t know how they handle it all the way from the source up to our households. However, since I haven’t experienced any diarrhea case in my household, it could be safe” (FGD 1, female 42 years old).***

Despite the fact that the quality of drinking water in many households was questionable, HWT was not preferred by some participants. One specific comment that was said to be a great hindrance to HWT was;

***“Although I am not sure of safety of the drinking water quality, I don’t like treating it because it loses its original natural taste especially when chlorinated” (FGD 3, female 22 years).***

#### 4.7.2 Causes of poor water quality

It was noted from public health officers (PHOs) who were interviewed that drinking water sample collection from households was a rare occurrence with major focus being on water sources. This is a comment from a PHO in a KII concerning factors contributing to water quality at the source;

***“Most of the water sources are contaminated because of close proximity to sanitary facilities and some tenants discharge raw sewage to rivers during the night. Unless water is from a piped system or treated in household, the water is not safe for drinking” (KII 1, male 50 years).***

Participants from GWASCO who took part in the KIIs seemed to agree that fecal contamination of drinking water is a possible phenomenon not only within households but also along the distribution network.

***“We sometimes experience bursts and leakages on sewer line which run parallel to water pipes and this could lead to water contamination” (KII 2, Female 34 years).***

## 5 Discussion

In more than half (65.4%) of the sampled households, household heads were males. The same trend was also noted in terms of marital status whereby 63.7% of the household heads were married. Also, more than half of the households had less than 5 members.

In this study, there was no direct significant relationship between socio-demographic features and drinking water quality. Similar findings were noted by a related study in Ghana (Boateng et al., 2013) whereby all the studied socio-demographic characteristics had no relationship with household water quality as indicated by a bivariate analysis. The findings are however contrary to a study by in Zimbabwe by Rameck (2018) which found out that there existed an association between drinking water quality and education levels of household head. A study carried out in Bomet municipality, Kenya (Koskei et al., 2013) also found out that there existed a strong association between education and occupation of household head and type of water source used.

Findings from this study showed that there existed a relationship between family size and contamination of drinking water with total coliforms. In another study conducted in Pakistan by Rauf and others (Rauf et al., 2015), related results were noted whereby; household size determined the choice of drinking water source which in turn affects the water quality. In their study, smaller and wealthier households were more likely to use improved water sources which are generally associated with safer drinking water. Similarly, a study by Almah and others (Armah et al., 2018) found out that small size households are more likely to obtain drinking water from improved sources and have access to improved sanitation than medium and large sized households. It has also been noted that households with many members have higher consumption and more expenditure especially in water bills (Simelane et al., 2020).

Three quarters of the studied households were not practicing any method of water treatment. According to a study carried out earlier in Kisii County by Misati (A. G. Misati, 2016), 58% of households never treat their drinking water. Such low level of HWT practice was also noted by Kurui and others (Kurui et al., 2019) who found out that only 34% of households surveyed used home water treatment techniques. Similarly, according to a study in Pakistan (Anwer et al., 2011), 73% of households were not using any water treatment technique. For those who practiced it, majority (44.6%) applied boiling because it is the cheapest measure of improving water quality at household level.

Drinking water source and quality perception were found have a significant statistical relationship with HWT. A large proportion of households that obtained their drinking water from unimproved sources treated their drinking water with well water users recording the highest percentage of 62.5%. Majority (72.9%) of the households that perceived their drinking water to be unsafe practiced HWT while only 11.2% of those that perceived it to be safe practiced the same. In another study in Puerto Rica to establish the perception and socio-demographic factors associated with household drinking water management strategies, it was found out that water treatment was more likely in households that believe that their water is of low quality (Jain et al., 2014). A similar study in South-Western Uganda by Saturday (2016) also found out that majority of those who never treat their drinking water perceive it to be safe while others mentioned factors such as bad taste and smell of treated water.

The study found a strong relationship between fecal contamination of household drinking water and the following factors; water source, transportation container, covering during transportation, storage container, method of drawing from storage container, feces disposal and garbage disposal method. A similar study in Zimbabwe (Rameck, 2018) came up with similar results whereby drinking water quality was significantly associated with type of toilet facility, handwashing practices, water source, transportation container, covering and method of drawing from the storage container. Other studies that came up with similar findings include Agensi et al. (2019), and Mudau et al. (2017), which found out that the type of storage container as well as covering of the container greatly influenced the quality of household drinking water. A study by Usman et al. (2016) however noted that water in narrow mouthed container were as well prone to contamination due to difficulty in cleaning their inner surface. They therefore tend to harbor bacteria over time. Frequent cleaning and shorter storage period is therefore necessary to ensure water safety.

## 6 Conclusion

Most of the studied hygiene and sanitation practices were found to be having strong relationship with household drinking water quality. These include; water source, transportation container, covering during transportation, storage container, method of drawing from storage container, feces disposal and garbage disposal method. This means that safe water from the source is likely to become contaminated in the household. That is the reason why HWT is highly recommended. However, in this study HWT was very low in Kisii Town with only 25% of the sampled households practicing it. Factors that were found to have a strong relationship with HWTS are drinking water source and water quality perception. Post contamination after HWT could also occur and therefore this practice should be accompanied by good hygiene and sanitation practices.

## 7 Recommendation

• Exposed solid wastes were highly associated with unsafe household drinking water. This could be because it attracts houseflies which are the key transmission agents for fecal-oral diseases. The Kisii Town council should therefore ensure that the wastes at different collection sites are collected daily and taken to a designated dumping site.
• Exposed excreta were also associated with fecal contamination of drinking water. Open defecation free (ODF) campaigns by public health officers should be reinforced to ensure that the unimproved pit latrines have squat-hole covers.
• Water quality should be included as a topic of concern during health education and promotion activities to educate people on the importance of good hygiene and sanitation practices as well as HWT. The residents should be educated on the importance covering water transportation and storage containers, using narrow mouthed containers and frequently cleaning it.

## 8 Ethical consideration

The research was permitted by the National Commission for Science, Technology and Innovation after obtaining ethical approval from Kenyatta University Ethical Review Committee. Participants were issued with informed consent forms which they signed having understood it fully before commencement of data collection. They were informed that participation was voluntary and deciding to withdraw from the study/interview at any point could not be penalized.

## 9 Limitation of the study

Water handling practices were studied by interviewing household heads in the study area. These included the period between fetching at the source until consumption in the household. The sanitation practices around the water sources were however not studied to ascertain factors that could lead to their contamination.

## Declarations

### Author contribution statement

J.K. Ondieki & Omanga Kenyanya: Conceived and designed the experiments; Performed the experiments; Analyzed and interpreted the data; Wrote the paper.

D.N. Akunga & P.N. Warutere: Conceived and designed the experiments; Analyzed and interpreted the data.

### Funding statement

This research did not receive any specific grant from funding agencies in the public, commercial, or not-for-profit sectors.

### Data availability statement

The data that has been used is confidential.

### Declaration of interest’s statement

The authors declare no competing interests.

### Additional information

No additional information is available for this paper.
